# Estimating 3D kinematics and kinetics from virtual inertial sensor data through musculoskeletal movement simulations

**DOI:** 10.3389/fbioe.2024.1285845

**Published:** 2024-04-02

**Authors:** Marlies Nitschke, Eva Dorschky, Sigrid Leyendecker, Bjoern M. Eskofier, Anne D. Koelewijn

**Affiliations:** ^1^ Machine Learning and Data Analytics Lab, Department Artificial Intelligence in Biomedical Engineering (AIBE), Friedrich-Alexander-Universität Erlangen-Nürnberg (FAU), Erlangen, Germany; ^2^ Institute of Applied Dynamics, Department of Mechanical Engineering, Friedrich-Alexander-Universität Erlangen-Nürnberg (FAU), Erlangen, Germany; ^3^ Institute of AI for Health, Helmholtz Zentrum München—German Research Center for Environmental Health, Neuherberg, Germany

**Keywords:** biomechanic analysis, inertial measurement units, wearable sensing, trajectory optimization, musculoskeletal model, change of direction

## Abstract

Portable measurement systems using inertial sensors enable motion capture outside the lab, facilitating longitudinal and large-scale studies in natural environments. However, estimating 3D kinematics and kinetics from inertial data for a comprehensive biomechanical movement analysis is still challenging. Machine learning models or stepwise approaches performing Kalman filtering, inverse kinematics, and inverse dynamics can lead to inconsistencies between kinematics and kinetics. We investigated the reconstruction of 3D kinematics and kinetics of arbitrary running motions from inertial sensor data using optimal control simulations of full-body musculoskeletal models. To evaluate the feasibility of the proposed method, we used marker tracking simulations created from optical motion capture data as a reference and for computing virtual inertial data such that the desired solution was known exactly. We generated the inertial tracking simulations by formulating optimal control problems that tracked virtual acceleration and angular velocity while minimizing effort without requiring a task constraint or an initial state. To evaluate the proposed approach, we reconstructed three trials each of straight running, curved running, and a v-cut of 10 participants. We compared the estimated inertial signals and biomechanical variables of the marker and inertial tracking simulations. The inertial data was tracked closely, resulting in low mean root mean squared deviations for pelvis translation (≤20.2 mm), angles (≤1.8 deg), ground reaction forces (≤1.1 BW%), joint moments (≤0.1 BWBH%), and muscle forces (≤5.4 BW%) and high mean coefficients of multiple correlation for all biomechanical variables (≥0.99) . Accordingly, our results showed that optimal control simulations tracking 3D inertial data could reconstruct the kinematics and kinetics of individual trials of all running motions. The simulations led to mutually and dynamically consistent kinematics and kinetics, which allows researching causal chains, for example, to analyze anterior cruciate ligament injury prevention. Our work proved the feasibility of the approach using virtual inertial data. When using the approach in the future with measured data, the sensor location and alignment on the segment must be estimated, and soft-tissue artifacts are potential error sources. Nevertheless, we demonstrated that optimal control simulation tracking inertial data is highly promising for estimating 3D kinematics and kinetics for a comprehensive biomechanical analysis.

## 1 Introduction

Kinematic and kinetic analysis of walking and running leads to essential insights in sport science and medicine. State-of-the-art measurement systems use marker-based optical motion capture combined with floor-embedded force plates or an instrumented treadmill. However, these systems are expensive and restricted to a controlled lab environment. Portable systems, such as RGB cameras or inertial sensors, can measure human motion outside of the lab and are far less expensive (Tan et al., 2023). Hence, portable measurement systems could enable longitudinal measurements of larger study populations under real-life conditions on the sports field, in the clinic, or at home. For example, change of direction movements could be measured during practice and in games to investigate the effectiveness of anterior cruciate ligament (ACL) injury prevention training programs with a larger evidence (Alanen et al., 2021) or osteoarthritis progression could be predicted based on joint loads during daily-living (Bennell et al., 2011).

Recently, the open-source web application OpenCap has been published, which estimates 3D human movement kinematics and kinetics from video data of two or more smartphones (Uhlrich et al., 2023). Pose estimation algorithms, like OpenPose (Nakano et al., 2020; Cao et al., 2021), directly extract the position of joints or body landmarks from the video data. OpenCap reconstructs the motion from these key points using inverse kinematics and obtains the kinetics by tracking the motion in an optimal control simulation of a musculoskeletal model (Uhlrich et al., 2023). Camera-based systems are promising solutions for large-scale studies outside of the lab. However, cameras require a line of sight, have a limited field of view, and are not privacy-preserving. These limitations make cameras unsuitable for the analysis, for example, of sports disciplines where the athlete travels long distances, team sports where other players cause occlusions, or home monitoring where the patient’s privacy should be preserved.

Inertial sensors are small, lightweight wearable sensors that have an unlimited capture volume compared to cameras. Hence, they can measure motion in an unrestricted environment without requiring a line of sight. However, inertial sensors do not measure absolute positions or joint angles but contain an accelerometer and gyroscope measuring linear acceleration and angular velocity (Hafer et al., 2023). This makes the motion reconstruction challenging since strapdown integration of noisy acceleration and angular velocity signals leads to drift (Weygers et al., 2020). Sensor fusion algorithms constraining the motion with kinematic models can improve the estimation of the joint kinematics as these models reduce integration drift (Roetenberg et al., 2013; Al Borno et al., 2022; Lavikainen et al., 2023). In addition to the kinematics, kinetics, specifically joint moments, ground reaction forces (GRFs), and muscle forces, give valuable information about the underlying mechanisms in the body. After motion reconstruction, the kinetics can be estimated with inverse dynamics (Karatsidis et al., 2019) using estimated GRFs (Skals et al., 2017) or using pressure insoles (Wang et al., 2023). However, this approach has the disadvantage that multiple processing steps are required, while it also results in inconsistencies between kinematics and kinetics.

Alternatively, machine learning is a promising method to map inertial measurements to kinematic or kinetic variables [see Gurchiek et al. (2019); Lee and Lee (2022) for reviews]. Trained machine learning models can be applied in real-time, allowing direct feedback to the user. However, many machine learning models do not provide a complete analysis but estimate only a small number of variables for a specific application, for example, vertical GRF and knee flexion angle (Wouda et al., 2018) or knee flexion and adduction moments (Stetter et al., 2020). Moreover, separate machine learning models were trained for kinematics and kinetics without taking the physical relation of the estimated variables into account (Mundt et al., 2021; Hossain et al., 2023). These drawbacks make machine learning models poorly generalizable to other applications and hinder explainability of potential biomechanical findings.

Physics-based simulations, specifically open-loop optimal control simulations of musculoskeletal models, have been investigated extensively as a tool to reconstruct joint and muscle kinematics and kinetics from optical motion capture and GRF measurements (e.g., van den Bogert et al., 2011). A simulation is generated by solving an optimal control problem that minimizes a multi-objective function combining a tracking term and an energy-related term with respect to the system dynamics of the model. As a result, the estimated variables are mutually consistent, enabling the interpretation of causal chains such that a certain joint angle change can be related to a change in joint moments or muscle forces. However, solving these optimal control problems, especially for complex 3D full-body musculoskeletal models, requires computationally efficient algorithms (Nitschke et al., 2020).

We previously showed that optimal control simulations of a sagittal-plane lower-body musculoskeletal model could accurately estimate kinematics and kinetics from inertial data of seven sensors for straight walking and running with Pearson correlation coefficients greater than or equal to 0.93 and 0.90 for the kinematics and kinetics, respectively (Dorschky et al., 2019). Furthermore, we found that the same approach achieved similar accuracy using sparse sensor setups, i.e., setups without an inertial sensor on each body segment of interest (Dorschky* et al., 2023). The optimal control problems of both studies tracked the mean accelerometer and gyroscope signals of multiple gait cycles by minimizing the sum of squared differences between the mean measured and simulated signals normalized by the variance of the measured signals (Dorschky et al., 2019; Dorschky et al., 2023). The normalization helps not to track signal components that are not reproducible over multiple gait cycles. Additionally, a task constraint enforced the simulation to generate periodic straight gait cycles to improve convergency. Accordingly, this research was limited to 2D lower-body kinematics and kinetics of straight walking and running.

**FIGURE 1:**
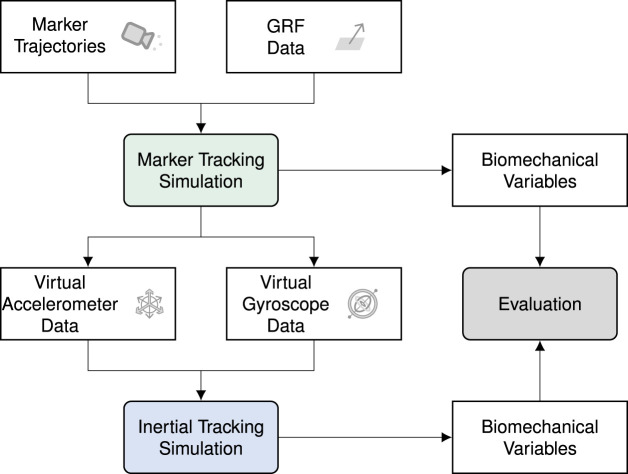
Processing pipeline. We used marker tracking simulations that tracked marker trajectories from optical motion capture and ground reaction force (GRF) data as reference (Nitschke et al., 2023b). Then, we computed virtual accelerometer and gyroscope data which we tracked in inertial tracking simulations. In the evaluation, we compared various biomechanical variables of the inertial tracking simulations to those from the marker tracking simulations.

In this work, we extended the previous research on optimal control simulations for the reconstruction of kinematics and kinetics from inertial data to the application for arbitrary 3D running movements, including change of direction movements. Our contribution to optimal control simulations tracking inertial data is three-fold: (1) We simulated 3D full-body musculoskeletal models leading to a high number of unknowns in the optimization problem and, therefore, a high complexity; (2) We reconstructed individual trials instead of tracking the mean of multiple trials normalized by the variance allowing us to investigate changes between trials; (3) We did not apply any task constraint or assumed any initial state to be able to reconstruct arbitrary running movements without any prior knowledge about the movement. To investigate if creating such reconstructions is theoretically feasible, i.e., if the inertial sensor data without an initial state contains sufficient information about the kinematics and kinetics, we reconstructed movements from virtual inertial sensor signals, for which the desired solution was known exactly. Virtual data allows us to evaluate our proposed approach in the absence of potential sources of errors, such as noise or soft-tissue artifacts. As a reference, we used optimal control simulations, which tracked marker trajectories from optical motion capture and GRF data (Nitschke et al., 2023b). From these marker tracking simulations, we computed virtual accelerometer and gyroscope data. To evaluate, we generated inertial tracking simulations from the virtual inertial data for three trials each of straight running, curved running, and a v-cut of 10 participants. Finally, we compared the inertial signals, pelvis translation, angles, GRFs, joint moments, and muscle forces of the marker and inertial tracking simulations (see Figure 1).

## 2 Methods

### 2.1 Data set

We used optical motion capture data of 10 healthy young participants (4 female, 6 male; age: 27.5 ± 3.5 years; height: 1.76 ± 0.10 m; mass: 71.3 ± 12.1 kg) (Nitschke et al., 2022). The recordings contained trajectories of 42 reflective markers recorded with 11 infrared cameras (175 Hz, Qualisys, Gothenburg, Sweden) and GRFs of the right and left foot (1,750 Hz, Bertec Corporation, Columbus, United States). The participants first performed a static trial in a neutral pose with the palms pointing towards the body. Afterward, the participants performed multiple trials of straight running, curved running with a radius of 7 m, and a 90 deg v-cut.

**FIGURE 2:**
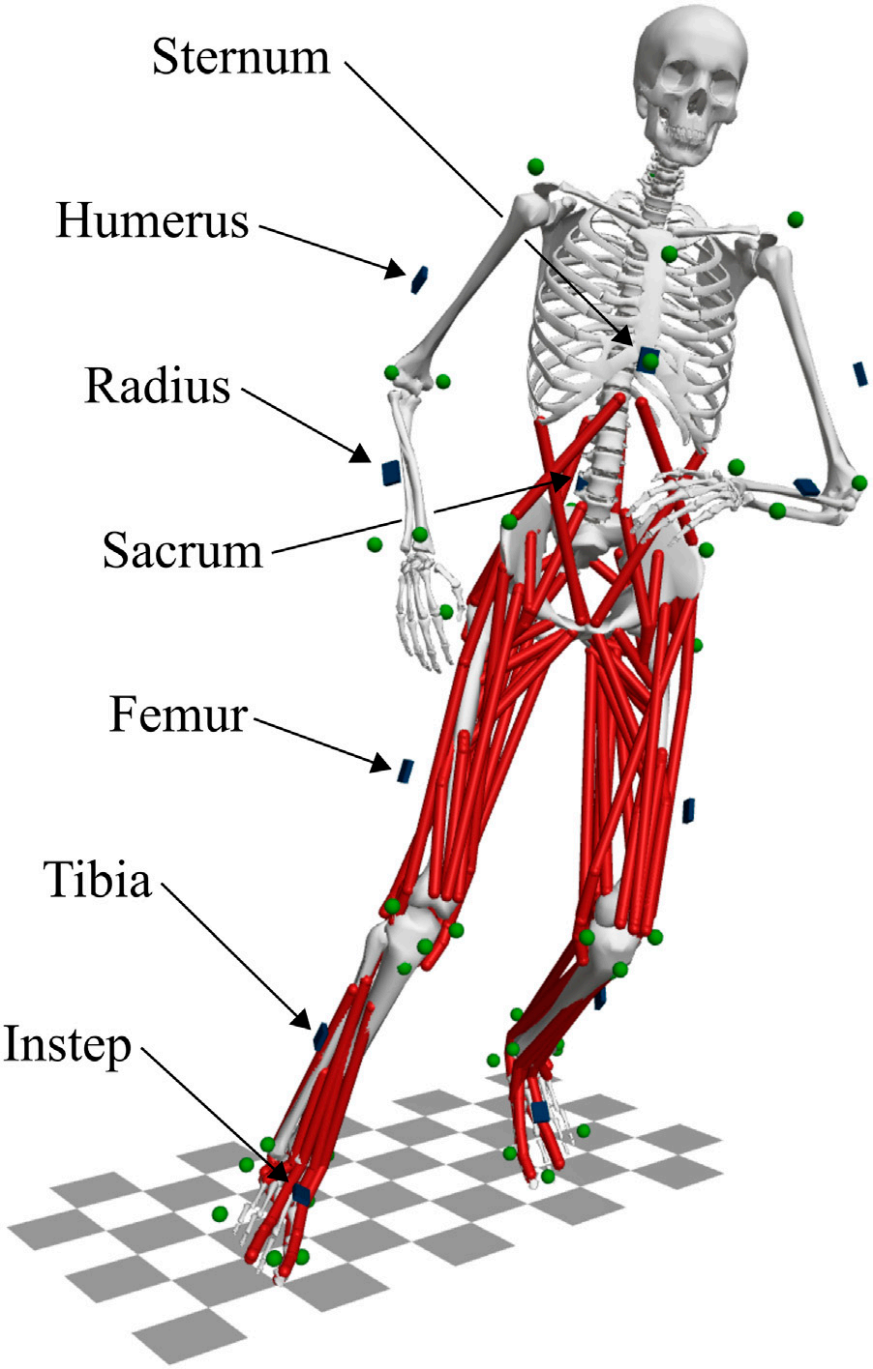
Musculoskeletal model “runMaD” (Nitschke et al., 2020) illustrating the placement of the inertial sensors shown in blue and markers in green. In total, 12 virtual inertial sensors were placed on the sternum, sacrum, laterally on the right and left humerus, radius, femur, and tibia, and on the right and left instep (fixed to the calcaneus). Data from 42 reflective markers attached to anatomical landmarks were used as a reference.

We have previously used the optical motion capture data to evaluate the reconstruction of change of direction motions by directly tracking marker and GRF data in 3D optimal control simulations (Nitschke et al., 2023b). Here, in the current study, we used the results of these marker tracking simulations as a reference. To generate virtual inertial sensor data, we applied an inertial sensor model (see Section 2.3) to the marker tracking simulations. We placed 12 virtual inertial sensors on the sternum, sacrum, laterally on the right and left humerus, radius, femur, and tibia, and on the right and left instep at the shoes (see Figure 2). To represent differences in sensor placement, we shifted the virtual sensors along the body segments by a defined offset for each participant. For example, for the first participant, all sensors were shifted by 0.5 cm along the body segments from the original sensor positions. For the 10 participants, we used offsets of ±0.5, ±1.0, ±2.0, ±3.0, or ±4.0 cm, respectively.

### 2.2 Musculoskeletal model

We used the 3D full-body musculoskeletal model “runMaD” (Nitschke et al., 2020) for the optimal control simulations. The model has been adapted for running motions with a change of direction and has 33 degrees of freedom, 92 muscle-tendon units in the lower body, and five torque actuators per arm (see Figure 2). For the simulation, we fitted polynomial functions to describe the muscle-tendon length depending on the joint angles and modeled ground contact with a penetration-based model with eight contact points at each foot [see supplemental information of Nitschke et al. (2020)]. The generic model was scaled with OpenSim 4.3 (Seth et al., 2018) using the marker trajectories of the static trial to match each participant (Nitschke et al., 2023b).

### 2.3 Inertial sensor model

**FIGURE 3:**
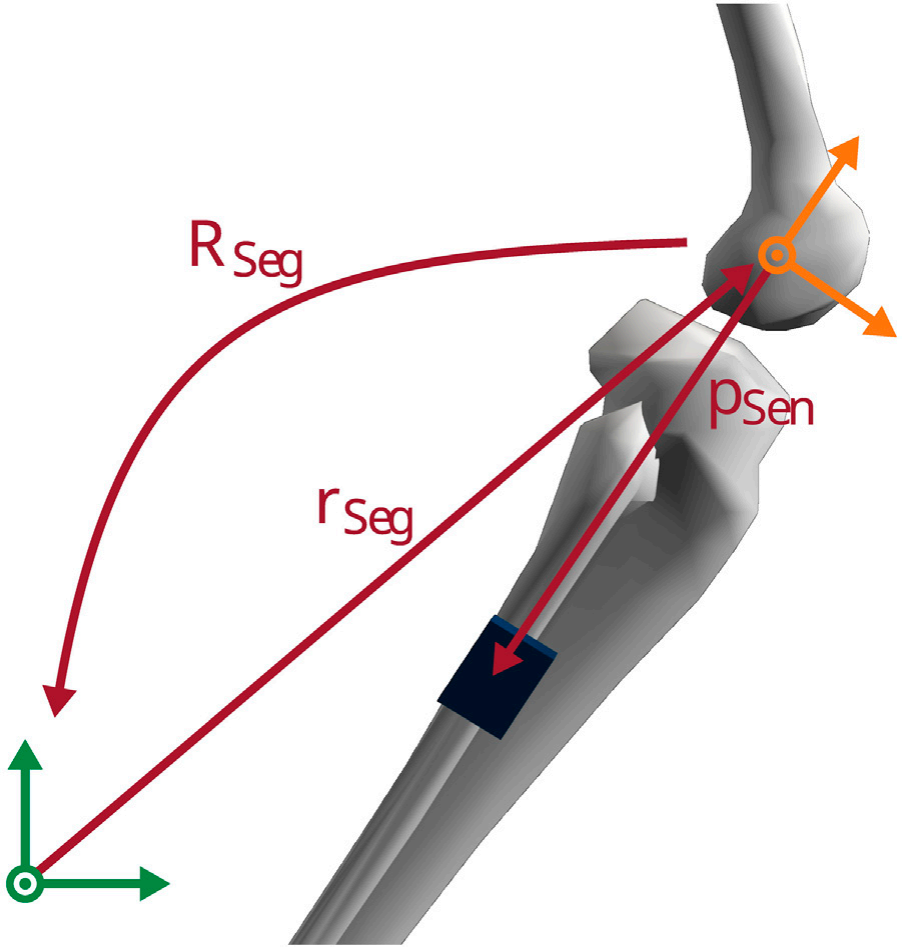
Representation of the inertial sensor model. The position of the virtual sensor **p**_*Sen*_ was defined in the segment coordinate system (orange). The position of the segment origin **r**_*Seg*_ and the segment orientation **R**_*Seg*_ were defined in the global coordinate system (green).

We employed an inertial sensor model to compute virtual acceleration and angular velocity for a given state trajectory of the musculoskeletal model. We used it first to generate virtual inertial data from marker tracking simulations and then to track this data in the optimal control problem. We assumed rigid attachment of the virtual sensor on the body segment at a known position **p**_*Sen*_ relative to the segment origin (see Figure 3). Furthermore, we assumed that the sensor and body segment axes were aligned. We obtained the position of the segment origin **r**_*Seg*_ and the segment orientation **R**_*Seg*_ in the global coordinate system using forward kinematics.

Using this information, we computed the virtual acceleration **a** as following (van den Bogert et al., 1996):

$$a=R_{\mathrm{Seg}}^{T}\left({\ddot{r}}_{\mathrm{Seg}}+{\ddot{R}}_{\mathrm{Seg}}p_{\mathrm{Sen}}-g\right),$$ (1)

where ${\ddot{r}}_{\mathrm{Seg}}$ and ${\ddot{R}}_{\mathrm{Seg}}$ denoted the second order derivatives of the segment origin and orientation, respectively. The global gravity vector was $g={\left(0,-9.80665,0\right)}^{T}$ . Furthermore, we obtained the virtual angular velocity $\omega ={\left(\omega_{x},\omega_{y},\omega_{z}\right)}^{T}$ using the following skew-symmetric matrix (Craig, 2005):

$${\left[\omega \right]}_{\times }=R_{\mathrm{Seg}}^{T}{\dot{R}}_{\mathrm{Seg}}=\left(\begin{array}{ccc} 0 & -\omega_{z} & \omega_{y}\\ \omega_{z} & 0 & -\omega_{x}\\ -\omega_{y} & \omega_{x} & 0 \end{array}\right).$$ (2)

Additionally, we defined the analytical derivatives of the acceleration **a** and the angular velocity ***ω*** to solve the optimal control problem. The same model implementation was used to generate virtual inertial data and to track this data. Hence, theoretically, our virtual inertial data could be tracked exactly, such that the resulting simulation would match the reference motion exactly.

### 2.4 Optimal control problems

To reconstruct the movement from the inertial data, we generated inertial tracking simulations by solving optimal control problems using the scaled musculoskeletal models. We aimed to find control trajectories **u** that result in state trajectories **x**, i.e., a motion, matching the given inertial data as closely as possible while minimizing effort. To do so, we used direct collocation and backward Euler discretization to formulate a constrained non-linear optimization problem with the multi-objective function *J*(**x**, **u**) and the model dynamics **f** as constraints.

#### 2.4.1 Objective function

We defined the multi-objective function *J*(**x**, **u**) as a weighted sum of accelerometer tracking *J*_*acc*_, gyroscope tracking *J*_*gyr*_, muscular effort *J*_*mus*_, torque effort *J*_*eff*_, and a small regularization term *J*_*reg*_:

$$J\left(x,u\right)=\;W_{\mathrm{acc}}J_{\mathrm{acc}}\;+\;W_{\mathrm{gyr}}J_{\mathrm{gyr}}\;+\;W_{\mathrm{mus}}J_{\mathrm{mus}}\;+\;W_{\mathrm{tor}}J_{\mathrm{tor}}\;+\;W_{\mathrm{reg}}J_{\mathrm{reg}},$$ (3)

with the weights *W*_*acc*_, *W*_*gyr*_, *W*_*mus*_, *W*_*tor*_, and *W*_*reg*_.

The tracking terms minimized the squared difference between the tracked accelerations *a* or angular velocities *ω* and the estimated signals $\hat{a}$ or $\hat{\omega }$ as follows:

$$J_{\mathrm{acc}}=\frac{1}{\left(N-1\right)N_{\mathrm{acc}}}\;\overset{N-1}{\underset{k=1}{\sum }}\;\overset{N_{\mathrm{acc}}}{\underset{i=1}{\sum }}\frac{{\left(a_{i}\left[k\right]-{\hat{a}}_{i}\left[k\right]\right)}^{2}}{\sigma_{a,i}^{2}},$$ (4)

$$J_{\mathrm{gyr}}=\frac{1}{NN_{\mathrm{gyr}}}\;\overset{N}{\underset{k=1}{\sum }}\;\overset{N_{\mathrm{gyr}}}{\underset{i=1}{\sum }}\frac{{\left(\omega_{i}\left[k\right]-{\hat{\omega }}_{i}\left[k\right]\right)}^{2}}{\sigma_{\omega ,i}^{2}},$$ (5)

where *N* was the number of collocation points, *N*_*acc*_ the number of tracked acceleration signals, and *N*_*gyr*_ the number of tracked gyroscope signals. We normalized the squared difference by the variance $\sigma_{a}^{2}$ or $\sigma_{\omega }^{2}$ of the tracked signal over time to scale the tracking errors relative to each other. Alternatively to the variance, the tracking error could be scaled by the peak-to-peak amplitude of the reference signal. However, we observed that the peak-to-peak amplitude was sensitive to high-impact motions causing high signal peaks, for example, during initial contact. In contrast to the gyroscope signal, the acceleration signal was not tracked for the last node *k* = *N* since computing the second-order derivative of the segment position and orientation at node *N* using backward Euler discretization would require a node *N* + 1.

To resolve the muscle redundancy problem, the muscular effort term minimized the sum of cubed neural excitation *n*_*e*_ of all lower-body muscles weighted by the muscle volume *w*_*mus*_ to account for differences in muscle size as follows:

$$J_{\mathrm{mus}}=\frac{1}{NN_{\mathrm{mus}}}\;\overset{N}{\underset{k=1}{\sum }}\;\overset{N_{\mathrm{mus}}}{\underset{i=1}{\sum }}\frac{w_{\mathrm{mus,i}}}{\overset{N_{\mathrm{mus}}}{\underset{j=1}{\sum }}w_{\mathrm{mus,j}}}{\left(n_{e,i}\left[k\right]\right)}^{3},$$ (6)

where *N*_*mus*_ = 92 was the number of muscle-tendon units. Furthermore, we minimized the sum of squared torque controls *m* actuating the upper body as follows:

$$J_{\mathrm{tor}}=\frac{1}{NN_{\mathrm{tor}}}\;\overset{N}{\underset{k=1}{\sum }}\;\overset{N_{\mathrm{tor}}}{\underset{i=1}{\sum }}{\left(m_{i}\left[k\right]\right)}^{2},$$ (7)

where *N*_*tor*_ = 10 was the number of torque actuators. Finally, a small regularization term was added to support the convergence of the solver (Nitschke et al., 2023b).

#### 2.4.2 Model dynamics

We ensured the model dynamics in their implicit form $f\left(x,\dot{x},u\right)=0$ using the following constraints:

$$f\left(x\left[k+1\right],\frac{x\left[k+1\right]-x\left[k\right]}{h},u\left[k+1\right]\right)=0\;\forall k=1,\ldots ,N-1,$$ (8)

$$f\left(x\left[1\right],\frac{x\left[2\right]-x\left[1\right]}{h},u\left[1\right]\right)=0,$$ (9)

with *h* = *T*/(*N* − 1) and the duration *T* of the motion. This combination leads to constant derivatives of the state and control vectors between nodes *k* = 1 and *k* = 2, which creates a solvable problem without having to fix the initial state or introduce a task constraint (Nitschke et al., 2023b). As a result, arbitrary motions can be reconstructed. More details about the model dynamics are given in Nitschke et al. (2020).

### 2.5 Simulations

We created inertial tracking simulations for three trials each of straight running, curved running, and a v-cut for each of the 10 participants. In the optimal control problems, we tracked the virtual acceleration and angular velocity of 12 inertial sensors (see section 2.1) in the x-, y-, and *z*-direction sampled at 175 Hz, resulting in *N*_*acc*_ = *N*_*gyr*_ = 36. The tracking signal started 10 additional samples before the actual motion of interest, i.e., before the initial contact, to avoid artifacts in the motion of interest due to the constraint in Eq. 9 (Nitschke et al., 2023b). We set the duration *T* of the motion to the duration of the tracking data. To be comparable to the marker tracking simulations, which served as reference, we applied the same values for the weights *W*_*mus*_ = 1, *W*_*tor*_ = 10^–1^, and *W*_*reg*_ = 10^–3^ (Nitschke et al., 2023b). Then, we empirically determined the tracking weights *W*_*acc*_ = *W*_*gyr*_ = 10 based on the first participant’s simulations such that the magnitudes of the neural excitations matched those of the marker tracking simulations. We weighted the accelerometer and gyroscope tracking terms equally since they should be tracked with the same importance.

As the inertial tracking data does not contain any position information and, thus, does not specify the global position and orientation of the model in the horizontal plane, the objective value would be the same for two identical simulations that are shifted or rotated in the horizontal plane. In other words, the global horizontal pelvis position and rotation at the first node must be prescribed to define the optimal control problem uniquely. This initial state of the horizontal pelvis position and rotation could be chosen arbitrarily without prior knowledge of the motion, e.g., it could be set to zero. However, to be comparable to the marker tracking simulations, we set the global horizontal pelvis position and rotation at the first node equal to those of the marker tracking simulations. All other initial states, i.e., the global vertical position, the pelvis obliquity, the pelvis tilt, the joint angles, and the muscle activations, did not need to be prescribed, since they are uniquely defined by Eqs. 8, 9.

We solved the constrained non-linear optimization problems with IPOPT 3.12.2 (Wächter and Biegler, 2006). We set the convergence tolerance for the scaled nonlinear program (NLP) error to 10^–4^ and the maximum number of iterations to 2 ⋅ 10^4^. The initial guess was a standing simulation generated with marker tracking (Nitschke et al., 2023b). To reduce the risk of local optima, we solved the optimal control problem additionally based on nine variations of this standing solution created randomly with a standard deviation of 10%. We ran the optimization on a high-performance cluster to parallelize the 900 inertial tracking simulations using four cores for each simulation and a wall time of 24 h.

### 2.6 Evaluation

First, we evaluated the convergence and the required wall time of the simulations. For each running trial, we then evaluated the solution that converged and had the lowest objective value of the 10 simulations generated from the different initial guesses. We analyzed the motion of interest without the additional samples at the beginning, i.e., the reconstructed motion from right initial contact to right initial contact. We visually compared the reconstructed motion of the inertial and marker tracking simulations by animating the models. To evaluate how close the inertial data was tracked and how well biomechanical variables were estimated, we generated trajectory graphs of accelerations, angular velocities, pelvis translation, angles, GRFs, joint moments, and muscle forces of the inertial and marker tracking simulations. Furthermore, we computed the root mean squared deviation (RMSD) and the coefficient of multiple correlation (CMC) (Ferrari et al., 2010) for the inertial data and the reconstructed kinematics and kinetics for each trial to quantify the waveform difference and waveform similarity, respectively. Then, we obtained the mean and standard deviation of the RMSDs and, using Fisher’s Z-transform, the mean of the CMCs to summarize the results for each variable type (e.g., accelerations) and each motion (e.g., straight running). We scaled the GRFs and muscle forces to body-weight percent (BW%) and the joint moments to body-weight body-height percent (BWBH%).

## 3 Results

From the 900 inertial tracking simulations, 718 simulations converged, 154 simulations failed in the restoration phase of the optimization, seven simulations reached the maximum number of iterations of 2 ⋅ 10^4^, and 21 simulations exceeded the wall time of 24 h. The simulations converged on average after a wall time of 6 h 16 min, where the fastest and slowest simulation converged after 2 h 53 min and 21 h 08 min, respectively. In the following, we analyzed, for each of the 90 trials, the solution that converged and had the lowest objective value of the simulations generated from the different initial guesses. For each trial, at least one simulation converged.

**FIGURE 4:**
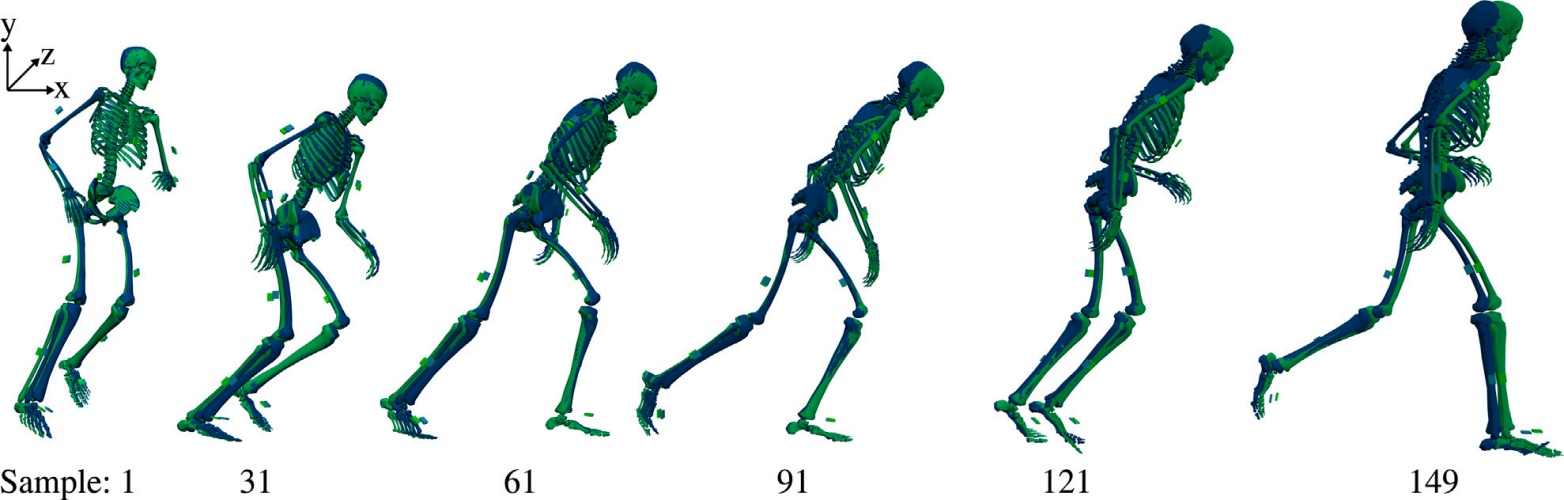
Kinematics for a v-cut including horizontal offset for visualization. The inertial and marker tracking simulation results are represented in blue and green, respectively. The simulations were generated with 175 Hz. The trial corresponds to trial three in Figures 5, 6 and to Figure 3 in Nitschke et al. (2023b).

Visual comparison of the animated motions showed that he inertial tracking simulations generally reconstructed the kinematics well, resulting in a natural running movement and a good overlay between the inertial and marker tracking simulations. Figure 4 highlights the good overlay for an exemplary v-cut since the two animations represent a similar movement pattern, resulting in closely overlapping skeletons. We also provide this visualization as a video in the supplemental data. However, the model ran slower for the inertial than the marker tracking simulations. This effect was small for most trials as the horizontal displacement of the pelvis at the end of the motion differed less than 10 cm to the marker tracking simulations (see Figures 4, 6). However, the effect was worse for 11 trials, leading to a difference in horizontal displacement of approximately 10 to 45 cm. The three worst trials had the highest objective values (>0.6) , the highest accelerometer tracking term ( >0.15 ; weighted), and the highest gyrosope tracking term ( >0.13 ; weighted) of all trials. However, the three worst trials had neither particularly low nor particularly high effort terms. Nevertheless, on average, the muscular effort term was smaller for the inertial tracking than for the marker tracking (0.06 vs. 0.09; weighted), meaning that muscles were less excited in the inertial tracking simulation than in the marker tracking simulation.

**FIGURE 5:**
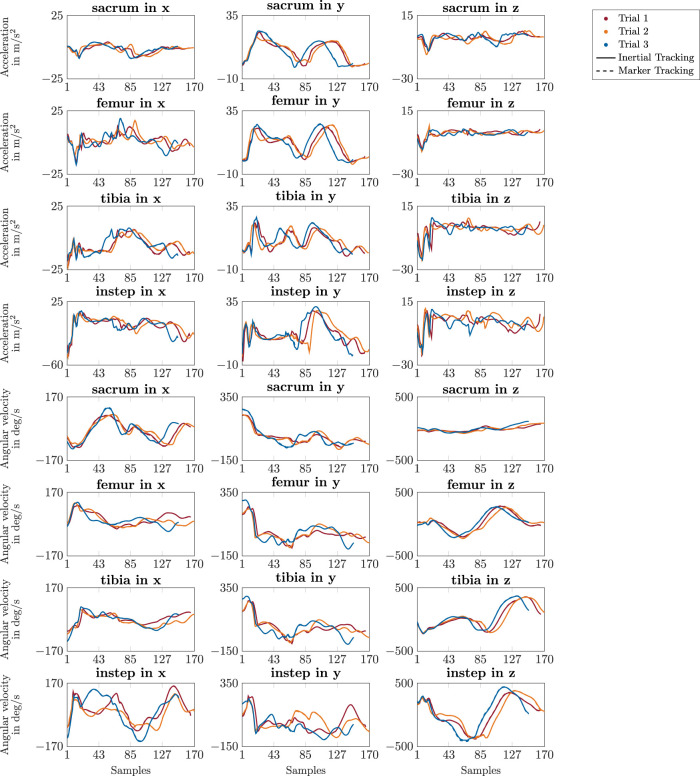
Trajectories of simulated acceleration and angular velocity data of the right side. The different colors represent the results of the three trials of a v-cut of one participant. The solid lines show the results of the inertial tracking simulations. The dashed lines show the reference obtained from the marker tracking simulations. The dashed lines of the marker tracking simulations are hardly visible as the inertial tracking simulations followed it almost perfectly. The sensor placement is illustrated in Figure 2. The simulations were generated with 175 Hz.

The inertial data, i.e., acceleration and angular velocity, was tracked closely in the inertial tracking simulations since differences compared to the reference signals of the marker tracking simulations were hardly visible in the trajectory plots (see Figure 5). This resulted in mean RMSDs of 0.2 m/s^2^ for the acceleration and 1.8 deg/s to 2.4 deg/s for the angular velocity signals for straight running, curved running, and v-cut (see Table 1), which corresponds to less than 0.3% of the maximum range of the tracked inertial signals. All mean CMCs were 1.0, with a minimum CMC value of 0.96. Only for the 11 trials for which the running motion was too slow the simulated inertial signals deviated considerably from the reference signals while still following the waveforms. For those 11 trials, the mean RMSDs increased to 0.6 m/s^2^ and 6.2 deg/s for acceleration and angular velocity, respecitvely. Furthermore, Figure 5 shows that although the inertial data of the different trials of a motion by the same participant had a similar waveform, they still differed distinctly.

**TABLE 1:** Mean ± standard deviation of the root mean squared deviation (RMSD) between inertial and marker tracking simulations. The results were summarized over all variables of the specific type (e.g., over all accelerations) and all trials of the respective motions straight running (SR), curved running (CR), and v-cut (VC).

	Motion	RMSD
Acceleration	SR	0.2 ± 0.5 m/s^2^
	CR	0.2 ± 0.4 m/s^2^
	VC	0.2 ± 0.2 m/s^2^
Angular velocity	SR	2.4 ± 6.7 deg/s
	CR	2.3 ± 6.0 deg/s
	VC	1.8 ± 2.2 deg/s
Translation	SR	20.2 ± 44.2 mm
	CR	14.0 ± 22.0 mm
	VC	8.8 ± 7.3 mm
Angle	SR	1.8 ± 5.2 deg
	CR	1.8 ± 5.5 deg
	VC	1.7 ± 5.0 deg
GRF	SR	1.1 ± 1.5 BW%
	CR	1.1 ± 1.0 BW%
	VC	0.9 ± 0.6 BW%
Joint moment	SR	0.1 ± 0.3 BWBH%
	CR	0.1 ± 0.2 BWBH%
	VC	0.1 ± 0.1 BWBH%
Muscle force	SR	4.5 ± 6.5 BW%
	CR	4.2 ± 6.3 BW%
	VC	5.4 ± 6.8 BW%

**FIGURE 6:**
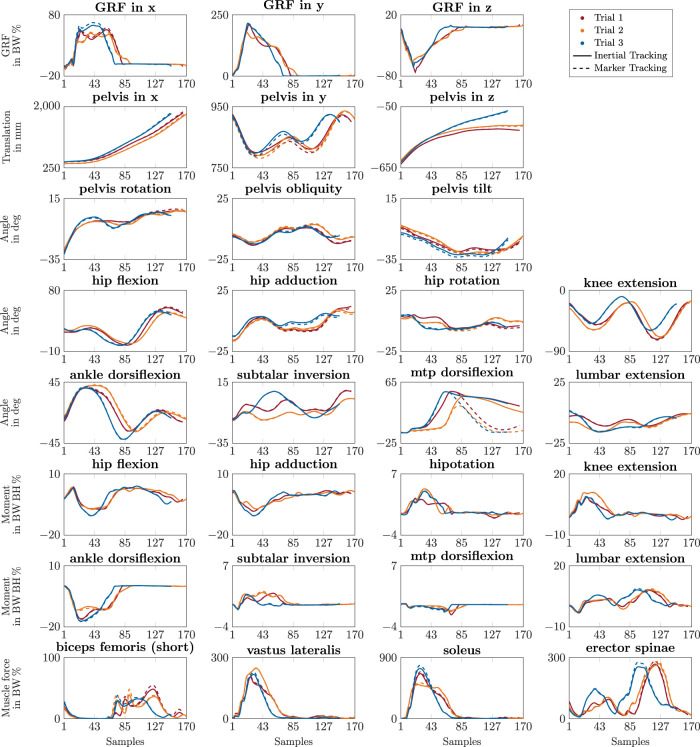
Trajectories of simulated kinematics and kinetics of the right side. The different colors represent the results of the three trials of a v-cut of one participant. The solid lines show the results of the inertial tracking simulations. The dashed lines show the reference obtained from the marker tracking simulations. The simulations were generated with 175 Hz.

The reconstructed kinematics and kinetics from the inertial tracking simulations, i.e., GRFs, translations, angles, moments, and muscle forces, mostly followed the reference from the marker tracking simulations closely. Differences between individual trials were reconstructed since characteristics of the reference variables are also present in the estimated variables (see Figure 6). For example, the peak ankle joint angle at push-off differs between the three trials in Figure 6, which is visible in the reference and reconstructed trajectories. Furthermore, the mean CMCs were always 0.99 or higher, although individual CMC values of some variables of some simulations, e.g., of the metatarsophalangeal (mtp) angle, were smaller or even zero. The mean RMSDs did not differ considerably between straight running, curved running, and v-cut (see Table 1). However, the mean RMSDs were larger for the translation of straight and curved running due to the 11 outliers with slow running. Even for those 11 trials, the reconstructed kinematic and kinetic trajectories roughly followed the reference signals, leading for those trials to mean RMSDs of 48.2 mm for translations, 3.0 deg for angles, 2.7 BW% for GRFs, 0.3 BWBH% for joint moments, and 7.2 BW% for muscle forces. Furthermore, the reconstructed mtp joint angles did not match the reference well, as the mtp was dorsiflexed during the swing phase. As a result, the RMSDs averaged over all simulations were 24.4 deg and 11.8 deg for the right and left mtp angle, respectively, while the mean RMSD of all other angles was 0.9 deg. In contrast to the mtp joint angles, the mtp joint moments achieved similarly high accuracies as the other joint moments. However, the mtp dorsiflexion muscles extensor digitorum and hallucis produced a considerably higher muscle force, and the mtp plantarflexion muscles flexor digitorum und hallucis produced a considerably lower muscle force for the inertial tracking than for the marker tracking during the swing phase.

The scaled models with the inertial sensor positions, the virtual accelerometer and gyroscope data, and the simulation results are provided online (Nitschke et al., 2023a).

## 4 Discussion

We investigated the feasibility of estimating 3D movement kinematics and kinetics from inertial sensor data using optimal control simulations. We found that individual repetitions of running motions can be reconstructed by tracking virtual accelerometer and gyroscope signals with a 3D full-body musculoskeletal model without the need for any prior knowledge about the running movement. The simulation facilitates a full biomechanical analysis by enabling the estimation of various mutually and dynamically consistent biomechanical variables, such as pelvis translation, angles, GRFs, joint moments, and muscle forces.

Optimal control simulations have multiple advantages compared to other approaches for motion reconstruction from inertial data. The linear acceleration and angular velocity signals can be tracked directly in the optimal control problem without applying any other sensor fusion algorithm, like the Kalman filter, to estimate sensor orientations. This is possible since the simulation takes the model dynamics into account and thus acts as a physical filter. Furthermore, the simulation uses the sensor position on the segment as input. Hence, it can handle different sensor placements in comparison to machine learning models, which are usually trained with predefined sensor locations. It has even been shown that the simulation can reconstruct sagittal-plane kinematics and kinetics of walking and running from a reduced number of inertial sensors (Dorschky et al., 2023). Moreover, GRFs are obtained in the simulation using a ground contact model, so they must not be measured or estimated separately while leading to consistency between reconstructed kinematics and kinetics. Therefore, the inertial tracking simulation can be valuable for many applications, especially when a comprehensive kinematics and kinetics analysis is required, without adapting the methodology or retraining a machine learning model. One possible application could be the analysis of ACL injury prevention in a natural environment where various biomechanical variables, like joint angles, joint moments, GRFs, and muscle activation, are from interest (Tan et al., 2023).

The inertial data was generally tracked closely, resulting in accurate kinematics and kinetics. The inertial tracking simulation could even reconstruct differences between trials of the same motion performed by one participant (see Figure 6). All in all, we obtained good RMSDs (see Table 1) in comparison to related work that compared reconstructed biomechanical variables from measured inertial data to measured optical motion capture data. For example, Karatsidis et al. (2019), compared to this work, reported mean RMSDs of 4.1 deg to 9.7 deg vs. 1.7 deg to 1.8 deg for joint angles, 2.1 BW% to 9.3 BW% vs. 0.9 BW% to 1.1 BW% for GRFs, and 0.3 BWBH% to 2.2 BWBH% vs. 0.1 BWBH% for joint moments. However, they used a step-wise approach of inverse kinematics and dynamics for inertial and optical motion capture, leading to inconsistencies between kinematics and kinetics.

While high accuracies were obtained for most joint angles, the simulation inaccurately estimated mtp joint angles during the swing phase. The mtp dorsiflexed during the swing phase due to higher muscle forces in the mtp dorsiflexion muscles and lower muscle forces in the mtp plantarflexion muscles compared to the marker tracking simulations. We found that this behavior was related to inaccuracies in the muscle-tendon parameters. The muscle-tendon parameters are such that a solution with dorsiflexed toes during the swing phase were more efficient than a solution with straight toes, because the muscular effort term in the optimal control problem promoted load sharing among the muscle-tendon units. The tracking data did not contain information for the ulna, talus, and toes since we did not place multiple sensors on the forearms or feet to increase usability. Therefore, this increased dorsiflexion did not cause the tracking term to increase. Nevertheless, optimal control simulations with a musculoskeletal model with more realistic muscle-tendon parameters should be able to predict the mtp angle without data of the toes since optimal control can also predict novel movements (Nitschke et al., 2020). We used scaled musculoskeletal models obtained from marker data using OpenSim (Seth et al., 2018), which alternatively could be generated from smartphone videos and OpenCap (Uhlrich et al., 2023) before using our proposed inertial motion capture in an unrestricted environment. However, these approaches scale only body segment parameters. In addition, the model parameters of the muscle-tendon units could be personalized to represent individual participants more accurately (Hicks et al., 2015).

Our study confirmed previously reported (Dorschky et al., 2019) convergency issues of IPOPT solving the optimal control problem. We solved the simulations from 10 different initial guesses to reduce the likelihood of the simulation ending up in a local optimum. However, this still seems to have happened for 11 out of the 90 trials. Nevertheless, we observed that the suboptimal trials’ objective values were clearly larger than the objective values of the other trials. Hence, instead of solving the problem for a fixed number of initial guesses, the problem could be solved with new initial guesses until the objective reaches a prior defined satisfaction criterion. This would avoid the risk of terminating in a suboptimal local optimum and would reduce the number of required solving processes in case a good solution was found early and would thus reduce computational cost. The initial guess is not the only factor influencing convergence, but also the solution algorithm (IPOPT) and its implementation could affect the final solution. While we implemented the gradient of the objective function and the Jacobian of the constraints analytically, we used a limited-memory quasi-Newton method to approximate the Hessian of the Lagrangian. Previous research showed that using the exact Hessian from algorithmic differentiation, rather than an approximation, led to a faster convergence for small pendulum simulations (Falisse et al., 2019). However, the opposite was observed for 2D predictive simulations of walking (Falisse et al., 2019). Furthermore, using the exact Hessian caused memory issues for 3D tracking simulations of walking (Falisse et al., 2019). Therefore, the solution process should be further investigated.

The optimal control problem contained a multi-objective function minimizing muscular effort and tracking error between the reference and simulated inertial data, meaning that the weighting between these two objectives needs to be defined. A high weighting of the effort term causes the simulation not to follow the reference data and to move at slower speeds. A high weighting of the tracking term leads to unrealistically fast activation and deactivation of the muscles. We determined the tracking weights empirically by matching the magnitudes of neural excitations with those observed in the marker tracking simulations for the first participant. In our results, most inertial tracking simulations slightly underestimated pelvis translation, possibly since effort can be minimized by moving less. The underestimated pelvis translation for inertial tracking is consistent with the result that tracking virtual inertial data yielded, on average, a lower muscular effort than the reference marker tracking simulations. A speed-related weight of the effort term could account for the effort increase required for movements at higher speeds (Dorschky et al., 2019). However, specifying a relation between effort and speed is more complex when simulating not only straight walking and running but arbitrary running motions. Moreover, the muscular effort term is likely also smaller for the inertial than for the marker tracking since virtual and no measured data was tracked. Theoretically, achieving a zero tracking error should be possible when tracking perfect virtual inertial sensor data, as we did in this work. Our inertial tracking simulation resulted in small tracking errors (see Table 1). In contrast, the marker tracking had to follow noisy measurements, causing a trade-off between effort and tracking.

Our work provides a proof of concept showing that it is feasible to reconstruct 3D full-body running kinematics and kinetics of individual trials by tracking virtual accelerometer and gyroscope data in an optimal control simulation of a musculoskeletal model. We tracked virtual data to refer to the exact solution during the analysis and to investigate whether the inertial data without prior knowledge about the initial state contains enough information to drive the simulations. In order to implement this method on actual measured data, we need to overcome some potential sources of errors. The virtual data was calculated from reference marker tracking simulations without modeling sensor noise. Therefore, we have not yet investigated the ability of our proposed method to handle the integration drift that is commonly associated with inertial sensor data (Weygers et al., 2020), which could be reduced when using a kinematic model (Roetenberg et al., 2013; Al Borno et al., 2022; Lavikainen et al., 2023). We also assumed a rigid connection of the sensor to the body segment while, in real measurements, the inertial sensor would move with respect to the bone resulting in soft-tissue artifacts. To accurately represent this behavior, a mass-spring model could be investigated for the connection of the inertial sensor. Furthermore, we assumed a known position and a perfect alignment of the sensor axes on the body segment the sensor was fixed to. For real measurements, the sensor-to-segment alignment would have to be assessed by applying estimation algorithms to defined functional calibration movements (Zrenner et al., 2020) or arbitrary movements (Seel et al., 2012). The sensor position on the segment could be measured manually (Dorschky et al., 2019) or obtained from an optimization algorithm (Seel et al., 2012).

While our feasibility study tracking virtual inertial data showed promising results compared to the literature, the accuracy has to be further evaluated in the context of specific applications. Being able to correctly reconstruct individual trials and thus estimate the movement variability of a participant is of high importance for many applications, like the assessment of fatigue (Cortes et al., 2014), the analysis of injury risk (Bartlett et al., 2007), or the investigation of injury prevention training programs (Alanen et al., 2021). We formulated the optimal control problem to reconstruct individual trials instead of a normalized mean of multiple trials. However, whether the method can detect statistically significant group differences must be analyzed for the respective application. Furthermore, our evaluation was limited to the analysis of 90 trials of 10 healthy young participants and to straight running, curved running, and a 90◦ v-cut. Although we formulated the optimal control problem to be capable of reconstructing arbitrary running motions, the accuracy should be reevaluated, especially if gait impairments are present.

In conclusion, we investigated musculoskeletal movement simulations for the reconstruction of 3D running kinematics and kinetics from inertial sensor data. Our results showed that it is feasible to reconstruct individual trials of arbitrary running motions with high accuracy by tracking virtual accelerometer and gyroscope data in an optimal control simulation of a 3D full-body musculoskeletal model. However, although optimal control simulations successfully reconstructed measured 2D inertial data (Dorschky et al., 2019) and despite our work on virtual 3D inertial data, tracking measured 3D inertial data of individual and arbitrary running motions remains an open challenge. Nevertheless, our work underlines that inertial tracking simulations are a promising tool for a comprehensive movement analysis outside of a motion lab.

## Data Availability

The datasets presented in this study can be found in online repositories. The names of the repositories and accession numbers can be found below: Optical motion capturing of change of direction motions reconstructed with inverse kinematics and dynamics and optimal control simulation (https://zenodo.org/records/6949012). 3D kinematics and kinetics of change of direction motions reconstructed from virtual inertial sensor data through optimal control simulation (https://zenodo.org/record/8183292).
