# Erratum: Estimating 3D kinematics and kinetics from virtual inertial sensor data through musculoskeletal movement simulations

**DOI:** 10.3389/fbioe.2024.1414850

**Published:** 2024-05-03

**Authors:** Frontiers Production Office

**Affiliations:** Frontiers Media SA, Lausanne, Switzerland

**Keywords:** biomechanic analysis, inertial measurement units, wearable sensing, trajectory optimization, musculoskeletal model, change of direction

Due to a production error, the Supplementary Material was not published.

The publisher apologizes for this mistake. The original version of this article has been updated.

