# Supplementary figures and images for: Estimating 3D kinematics and kinetics from virtual inertial sensor data through musculoskeletal movement simulations
Source: Front Bioeng Biotechnol. 2024 Apr 2;12:1285845. doi: 10.3389/fbioe.2024.1285845 (PMC11018991; doi:10.3389/fbioe.2024.1285845)

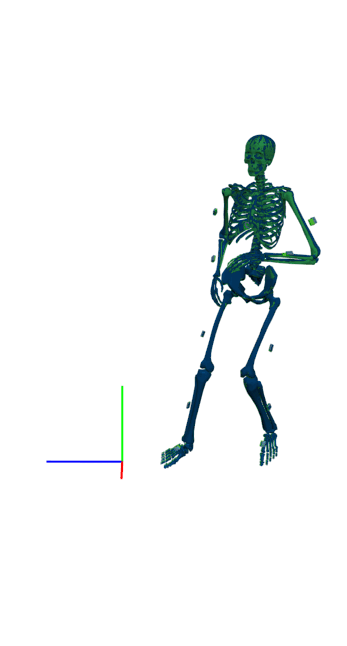

Supplement: Supplementary file 1 [file Presentation1.ZIP › Frontal_View_Every3rdSample_cropped.gif]

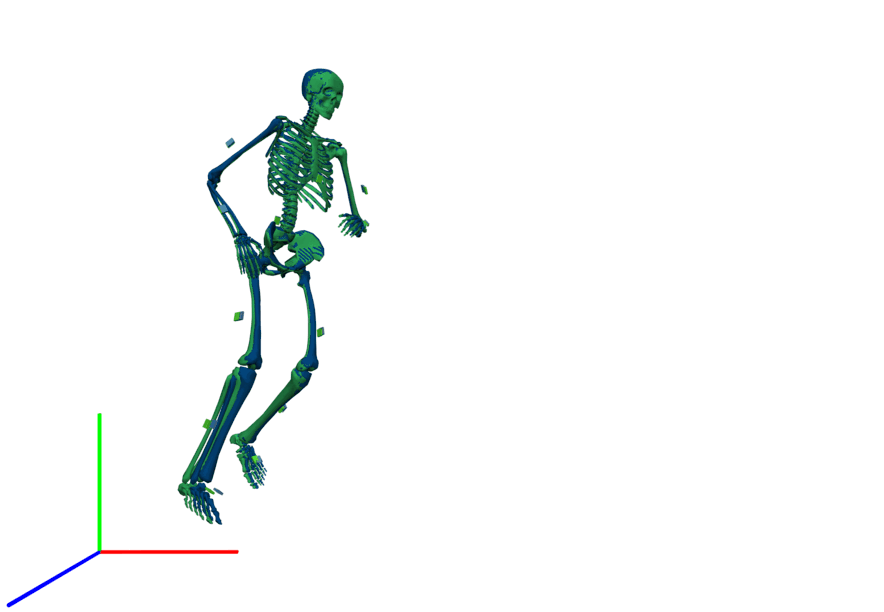

Supplement: Supplementary file 1 [file Presentation1.ZIP › Lateral_View_Every3rdSample_cropped.gif]
